# Posterior *HOX genes* and *HOTAIR* expression in the proximal and distal colon cancer pathogenesis

**DOI:** 10.1186/s12967-018-1725-y

**Published:** 2018-12-12

**Authors:** Fabiana Tatangelo, Annabella Di Mauro, Giosuè Scognamiglio, Gabriella Aquino, Antonio Lettiero, Paolo Delrio, Antonio Avallone, Monica Cantile, Gerardo Botti

**Affiliations:** 1 Pathology Unit, Istituto Nazionale Tumori Fondazione G. Pascale-IRCCS, Naples, Italy; 2 Colorectal Cancer Surgery Unit, Istituto Nazionale Tumori Fondazione G. Pascale-IRCCS, Naples, Italy; 3 Abdominal Oncology Unit, Istituto Nazionale Tumori Fondazione G. Pascale-IRCCS, Naples, Italy; 4 Scientific Direction, Istituto Nazionale Tumori Fondazione G. Pascale-IRCCS, Naples, Italy

**Keywords:** Proximal and distal CRC pathogenesis, HOX genes, Prognosis

## Abstract

**Background:**

Increasing evidences showed that the location of the primary tumor on the right (proximal) or left (distal) side of the colon have a prognostic/predictive value for colon cancer patients. However, the understanding of the molecular mechanisms that contribute to the pathogenesis in different location of colon is still unclear. Probably an important role could be played by genes that control the spatial–temporal development of bodily structures, such as HOX genes.

**Methods:**

The main purpose of this study was to analyze the expression of the paralogous 13 HOX genes and of the HOX regulating lncRNA *HOTAIR* in distal and proximal CRC cases. We have carried out a Tissue Micro Array with left and right CRC samples associated with all clinical-pathological parameters of patients. The expression of HOX genes was evaluated by immunohistochemistry and the staining of *HOTAIR* was performed by in situ hybridization using a specifically designed LNA probe.

**Results:**

All paralogous 13 HOX genes and *HOTAIR* are silent in normal tissue and expressed in CRC samples. *HOXB13, HOXC13* and *HOTAIR* showed a statistical association with lymph nodes metastasis (p value = 0.003, p value = 0.05, p value = 0.04). *HOXB13*, *HOXC13* and lncRNA *HOTAIR* are overexpressed in right CRCs samples (p value < 0 and p value = 0.021). *HOTAIR* is also strongly correlated with *HOXB13* (p value = 0.02) and *HOXC13* (p value = 0.042) expression.

**Conclusions:**

Our data highlighted an important role of posterior HOX genes in colorectal cancer carcinogenesis. Specifically, the aberrant expression of the *HOXB13*, *HOXC13* and *HOTAIR* in proximal colon cancers could add an important dowel in understanding molecular mechanisms related to tumor pathogenesis in this location.

**Electronic supplementary material:**

The online version of this article (10.1186/s12967-018-1725-y) contains supplementary material, which is available to authorized users.

## Background

Colorectal carcinoma (CRC) represents the third malignant tumor by incidence and mortality in Western countries. Recent data from American Society of Clinical Oncology (ASCO) have demonstrated significant anatomical site-specific disparities in colon cancer patients survival [1]. Patients with tumor in the left side (descending colon, sigmoid colon and rectum) survived longer than those with tumors originated on the proximal side (cecum and ascending colon). In addition, the location of the primary colon tumor would be able to predict survival and determine the optimal treatment choice for patients with metastatic colorectal cancer. In fact, tumors on the right side, which have an unfavorable prognosis, do not benefit significantly from the addition of Cetuximab, while those on the left side benefit from it [2].

Significant biological differences associated with different embryonic origin of colon (middle intestine for the proximal colon and the posterior intestine for the distal colon) were well documented [3]. Biological heterogeneity also reflects significant molecular differences. Numerous studies have shown that proximal cancers are more often associated with microsatellite instability (MSI) and may have mutations in *KRAS*, *BRAF* and *PIK3CA* genes [3, 4]. Distal tumors frequently showed a deletion of a chromosome 18q region, and an amplification of *EGFR* and *HER2* in 12% of cases [5].

However, the understanding of the molecular mechanisms that determine the pathogenesis of the tumor on the right or left side is still unclear. Probably an important role could be played by genes that control the spatio-temporal development of body structures. One of the most studied gene family and whose de-regulation is often associated with carcinogenesis and tumor progression is the Homeobox genes [6].

Homeobox genes are responsible for regulating normal embryonic development, cell differentiation and other critical processes of eukaryotic cell life. Several studies have shown that genes belonging to the class I homeobox genes (HOX genes) play a crucial role in neoplastic transformation in various human tissues. In particular, the genes belonging to HOX paralogous group 13 seem to carry out a relevant role in both tumor development and progression [7–13].

Recently it has been shown that the regulation of HOX gene expression is under the control of different non-coding RNAs (ncRNAs) some of which are present within HOX loci [14]. The most studied among them is a long non-coding RNA (lncRNA) named *HOTAIR* (Hox transcript antisense intergenic RNA) able to modulate metastatic progression in several human cancers [15].

Since some posterior genes of the HOX genes network, control the antero-posterior development of the gut [16], and their aberrant expression has been associated with colorectal carcinogenesis [17], in this study we decided to investigate the role of HOX genes belonging to the paralogous group 13 in a series of CRCs with different anatomical localization. We also focused the attention on the long non-coding RNA *HOTAIR* which controls *in trans* the expression of the locus HOX D genes and of several genes associated with metastatic progression [18].

## Methods

### CRCs patients

Eighty-two patients admitted to the National Cancer Institute “Giovanni Pascale” of Naples, between 2012 and 2017, were recruited in this study. All patients had provided written informed consent for the use of samples according to the institutional regulations and the study was approved by the ethics committee of the National Cancer Institute “G. Pascale”.

All CRCs cases have been reviewed by two pathologists (FT, GB) and graded and staged according to WHO 2010/AJCC 2017 classification criteria, on standard tissue sections.

Medical records have been reviewed for clinical information, including histologic parameters, assessed on standard H&E-stained slides, and tumor location (right or left).

### Immunohistochemistry analysis

All selected samples derived from formalin-fixed, paraffin embedded tissues (FFPE) including tumor, non-neoplastic colonic mucosa, and adenomatous dysplastic modifications areas. We have built a Tissue Micro Array (TMA) using cores representative of all three components. Immunohistochemical staining was carried out on slides from, (FFPE) in order to evaluate the expression of *HOX A13*, *HOX B13*, *HOX C13*, *HOXD13* and Carcinoembryonic antigen (CEA). Paraffin slides was then deparaffinized in xylene and rehydrated through graded alcohols. Antigen retrieval was performed with slides heated in 0.0.1 M citrate buffer (pH 6.0.) in a bath for 20 min at 97 °C. After antigen retrieval, the slides were allowed to cool. The slides were rinsed with TBS and the endogenous peroxidase has inactivated with 3% hydrogen peroxide. After protein block (BSA 5% in PBS 1×), the slides were incubated with primary antibody to human *HOX A13* (dilution 1:200, cod. Ab106503, Abcam, Cambridge, UK), *HOX B13* (dilution 1:300, cod. ab28575, Abcam, Cambridge, UK), *HOXC13* (dilution 1:1200, cod.ab55251, Abcam, Cambridge, UK), *HOX D13* (dilution 1:100, cod. Ab19866, Abcam, Cambridge, UK), anti-CEA antibody (dilution 1:100; Leica, Newcastle, UK) overnight. Sections were incubated with mouse anti-rabbit or goat anti-mouse secondary IgG biotinylated secondary antibody for 30 min. Immunoreactivity was visualized by means of avidin–biotin–peroxydase complex kit reagents (Novocastra, Newcastle, UK) as the chromogenic substrate. Finally, sections were weakly counterstained with haematoxylin and mounted.

### Evaluation of immunostaining

Antigen expression was independently evaluated by two experienced pathologists (FT/GB) using light microscopy. For paralogous 13 HOX genes nuclear and cytoplasmic localization were considered. All values of immunostaining were expressed only in percentage terms of positive cells. The percentage of positive cancer cells was evaluated in each sample by counting the number of positive cells over the total cancer cells in 10 non-overlapping fields using X400 magnification. Membrane and cytoplasmic CEA staining in tumor tissue was evaluated following an intensity score: negative (1), positive (2), or strongly positive (3).

### Probe design for In Situ Hybridization (ISH)

For the development of in situ hybridization method we designed an RNA probe of 20 bp. Specifically, we analyzed the sequence of *HOTAIR* (NCBI Reference Sequence: NC_000012.12). The gene is characterized by three different variants (variant 1: NR_047517.1; variant 2: NR_003716.3; variant 3: NR_047518.1) and for this reason we selected a sequence of approximately 20 bp common to all three variants. The sequence of the digoxin-labeled LNA™-Modified *HOTAIR* probe (Exiqon), is: 5DigN/TCTAAATCCGTTCCATTCCACT/3Dig_N.

### ISH analysis

*HOTAIR* expression was examined by in situ hybridization in CRCs paraffin-embedded sections by using a commercial kit (FFPE ISH Detection Kit Optimization DNA RNA (Exiqon) containing two control probes (“LNA scrambled” consists of the same sequence of the forward probe and “LNA U6 snRNA” containing the sequence u6 of small nuclear RNAs (snRNA), respectively positive and negative control of ISH method. The two control probes were used in each reaction to verify the correct execution of the method.

Briefly, after dewaxing and rehydration, the samples were digested with proteinase K, fixed in 4% paraformaldehyde and hybridized with a 5′ digoxin-labeled LNA™-modified *HOTAIR* probe (Exiqon) overnight at 55 °C. The samples were then incubated overnight at 4 °C with an anti-digoxin monoclonal antibody (Roche Applied Science). The sections were stained with nitro blue tetrazolium/5-bromo-4-chloro-3-indolylphosphate (NBT/BCIP) in the dark, mounted and observed. *HOTAIR*-positive staining (in blue) was detected on the membrane and in the cytoplasm of the cells. The staining scores were determined by microscopy on the basis of both intensity and proportion of HOTAIR-positive cells in 10 random fields under a 40× objective. The staining intensity of the cells was graded as previously reported [19]. The representative staining fields for each specimen were analyzed and scored independently by two observers who were blinded to each other and to the diagnoses of the specimens.

### Statistical analysis

The stratification of high/low expression was based on the median percentage of cells expressing the proteins (*HOXA13, HOXB13, HOXC13, HOXD13*) and the LncRNA *HOTAIR*.

The association between *HOX A13, HOX B13, HOX C13, HOX D13*, *HOTAIR* with the clinic-pathological data was conducted using the *χ*^2^ test considering the median of expression for each marker as cut-off. Cut-offs were schematized in Additional file 1: Table S1.

The Pearson *χ*^2^ test was used to determine whether a relationship exists between the variables included in the study. The level of significance was defined as *p *< 0.05. All the statistical analyses were carried out using SPSS statistics 20.

## Results

### Clinical-pathological features of CRCs patients

Selected patients and their clinical-pathological characteristics are summarized in Additional file 2: Table S2.

The age of patients ranged from 50 to 91 years, with an average age of 65 years. 56.1% were female and 43.9% were male. For 58.5% of CRCs patients the lesion was located in the proximal colon, while for 41.5% in distal colon. Regarding T stage, 3.7% of patients had a primary tumor located in submucosal (T1), 30.5% in muscularis propria (T2), for 51,2% the lesion extended beyond muscularis propria (T3), and for 7.3% invaded the peritoneal surface and/or adjacent organs (T4).

Regarding lymph nodal invasion, 64.9% of CRCs patients showed no lymph node metastases (N0), 21.6% had 1-3 lymph node metastases (N1), and 12.2% showed > 4 lymph node metastases (N2). The most of CRCs patients (99%) showed no distant metastases. For tumor grading stratification, 3.7% were well differentiated adenocarcinoma (G1), 90.2% were moderately differentiated adenocarcinoma (G2), and 6.1% poor differentiated adenocarcinoma (G3).

### Expression of paralogous 13 HOX proteins and HOTAIR mRNA in CRCs patients series

**Fig. 1:**
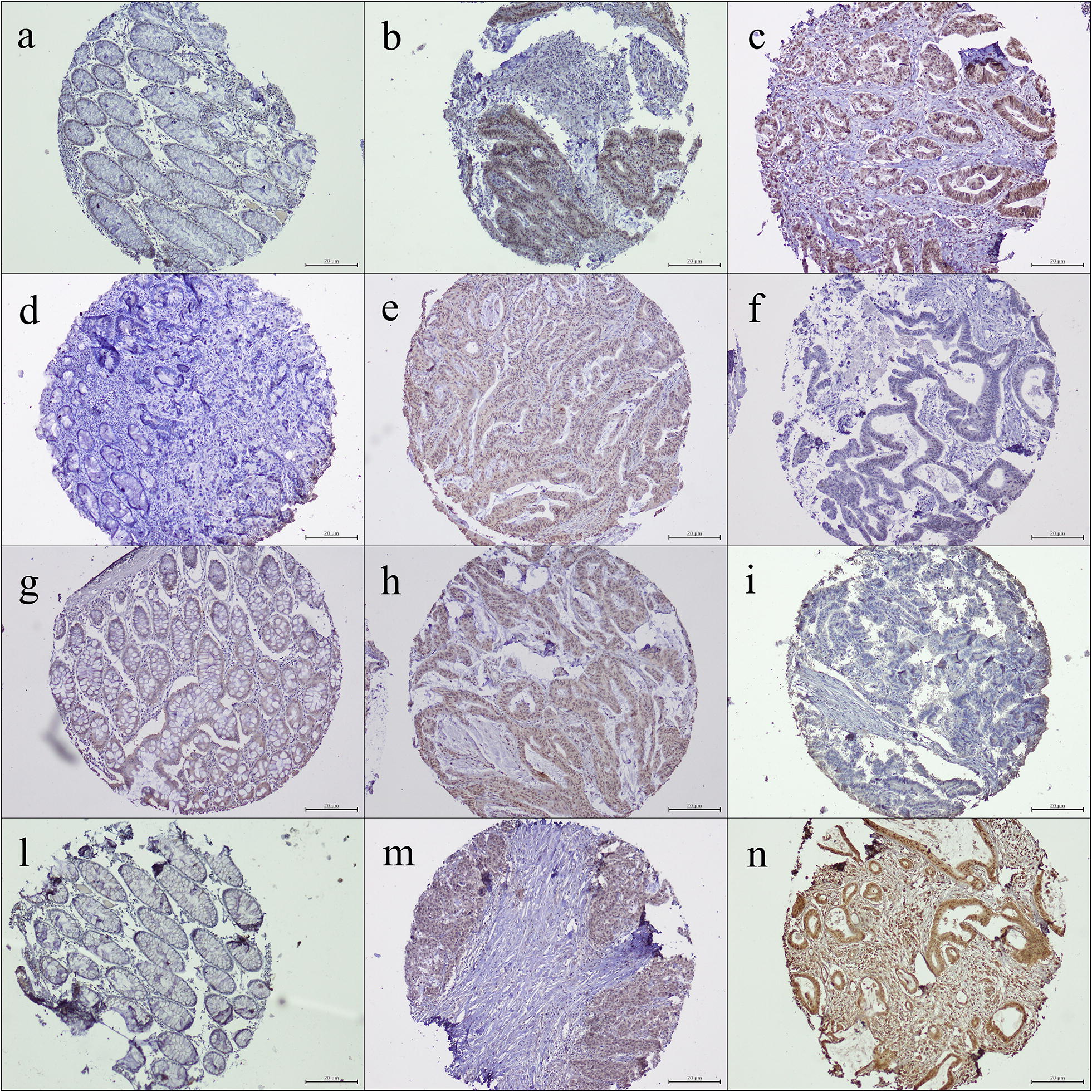
Paralogous group 13 *HOX* protein expression in colon tissue samples: **a** negative *HOX A13* expression in colonic mucosa (×20); **b** positive *HOX A13* nuclear expression in proximal CRC sample (×20); **c** positive *HOX A13* nuclear expression in distal CRC sample (×20); **d** negative *HOX B13* expression in colonic mucosa (×20); **e** positive *HOX B13* nuclear expression in proximal CRC sample (×20); **f** negative *HOX B13* nuclear expression in distal CRC sample (×20); **g** negative *HOX C13* expression in colonic mucosa (×20); **h** positive *HOX C13* nuclear expression in proximal CRC sample (×20); **i** negative *HOX C13* nuclear expression in distal CRC sample (×20); **l** negative *HOX D13* expression in colonic mucosa (×20); **m** positive *HOX D13* nuclear expression in proximal CRC sample (×20); **n** positive *HOX D13* nuclear expression in distal CRC sample (×20)

The immunohistochemical analysis mainly revealed a nuclear localization of paralogous 13 HOX proteins, whereas a cytoplasmic localization was observed only in some areas. *HOXA13, HOXB13, HOXC13* and *HOXD13* were not expressed in normal colonic tissues, whereas their expression was detected in the most of analyzed CRCs samples (Fig. 1).

**Fig. 2:**
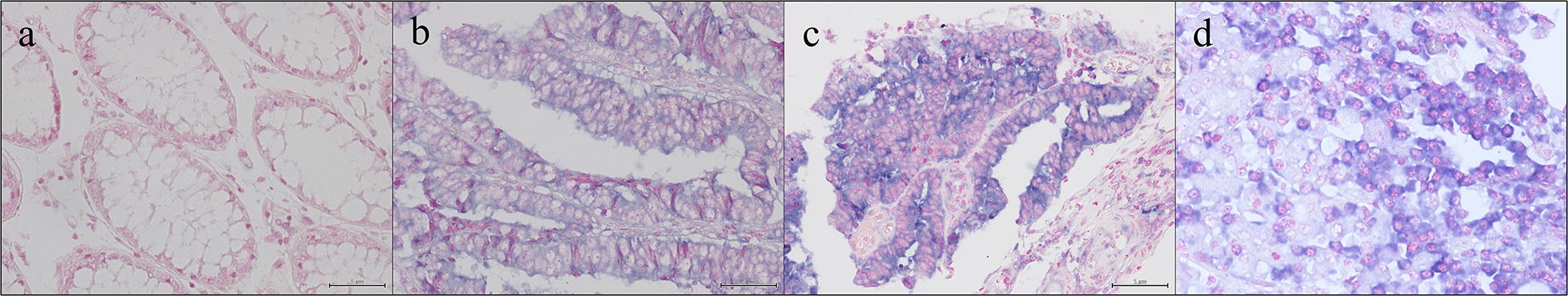
*HOTAIR* expression in colon tissue samples: **a** negative *HOTAIR* expression in colonic mucosa (×20); **b** positive *HOTAIR* cytoplasmic expression in adenomatous dysplastic area (×20); **c** positive *HOTAIR* cytoplasmic expression in CRC sample (×20); **d** detail of lymphocytes staining in tumor microenvironment of CRC sample (×60)

The same trend of expression was highlighted for the in situ determination of the lncRNA *HOTAIR*. HOTAIR appears to gradually increase in the transition from normal colonic tissue, adenomatous dysplastic area, adenocarcinoma, with a prevalent cytoplasmic and membrane staining in CRCs samples as shown in Fig. 2. In some cases *HOTAIR* staining was detected also in peri-intra tumoral lymphocyte component (Fig. 2).

### Relation between paralogous 13 HOX and HOTAIR expression with clinic pathological features of CRCs patients series

For the statistical elaboration we considered the median of expression for each marker as cut-off (low and high) (Table 1). In detail, *HOXA13 and HOXD13* appeared not correlated with clinic-pathological features of patients. *HOX B13* showed a trend of statistical association with sex and age, and a strong direct statistical association with lymph nodes metastasis (p value = 0.003). The same trend of association with lymph nodes metastasis was detected for *HOXC13* (p value = 0.051).

**Table 1:** Correlation between paralogous group 13 (*HOXA13, HOXB13, HOXC13, HOXD13*) and HOTAIR expression and main clinical features of CRC patients

	HOXA13		p-value	HOXB13		p-value	HOXC13		p-value	HOXD13		p-value	HOTAIR		p value
	Low	High		Low	High		Low	High		Low	High		Low	High	
Gender															
M	24 (29.6)	19 (23.5)	1	19 (23.2)	25 (30.5)	0.081	24 (29.3)	20 (24.4)	1	27 (32.9)	17 (21)	1	8 (10.0)	36 (45.0)	0.78
F	21 (25.9)	17 (21)		24 (29.3)	14 (17.1)		21 (25.6)	17 (20.7)		23 (29.1)	15 (20.8)		8 (10.0)	28 (35.0)	
Age															
< 50	18 (22.2)	18 (22.2)	0.5	23 (28.0)	13 (15.9)	0.078	18 (22)	18 (22)	0.5	20 (24.4)	16 (19.5)	0.494	11 (23.9)	12	0.057
> 50	27 (33.3)	18 (22.2)		20 (24.4)	26 (31.7)		27 (32.9)	19 (23.2)		30 (36;6)	16 (19.5)		4 (8.7)	19 (41.3)	
T															
T1/T2	15 (19.2)	16 (20.5)	0.36	17 (21.5)	14 (17.7)	1	15 (19.2)	16 (20.3)	0.25	19 (24.1)	12 (15.2)	1	6 (7.8)	25 (32.5)	0.04
T3/T4	28 (35.9)	19 (24.4)		26 (32.9)	22 (27.8)		30 (38)	18 (22.8)		29 (36.7)	19 (24.1)		10 (13.0)	36 (46.8)	
Grade															
G1	1 (1.2)	2 (2.5)	0.855	2 (2.4)	1 (1.2)	1	2 (2.4)	1 (1.2)	1	3 (3.7)	0	0.307	2 (2.5)	1 (1.25)	1
G2	41 (50.6)	32 (39.5)		38 (46.3)	36 (43.9)		40 (48.8)	34 (41.5)		45 (54.9)	29 (35.4)		40 (50)	32 (40)	
G3	3 (3.7)	2 (2.5)		3 (3.7)	2 (2.4)		3 (3.7)	2 (2.4)		2 (2.5)	3 (3.7)		3 (3.8)	2 (2.5)	
N															
N0	31 (41.9)	17 (20.7)	0.134	32 (43.2)	16 (21.6)	0.003	32 (44.4)	16 (21.6)	0.051	31 (41.9)	17 (20.7)	0.456	14 (19.4)	33 (45.5)	0.04
N+	11 (15.1)	14 (19.2)		8 (10.8)	18 (24.3)		11 (14.9)	15 (20.3)		14 (18.9)	12 (16.2)		2 (2.8)	23 (31.9)	

Regarding *HOTAIR* we detected a trend of statistical association with age of CRC patients (p value = 0.057) and a significant statistical association with T stage (p value = 0.04) and lymph nodes metastasis (p = 0.04).

### Relation between paralogous 13 HOX and HOTAIR expression and right/left CRC location

**Fig. 3:**
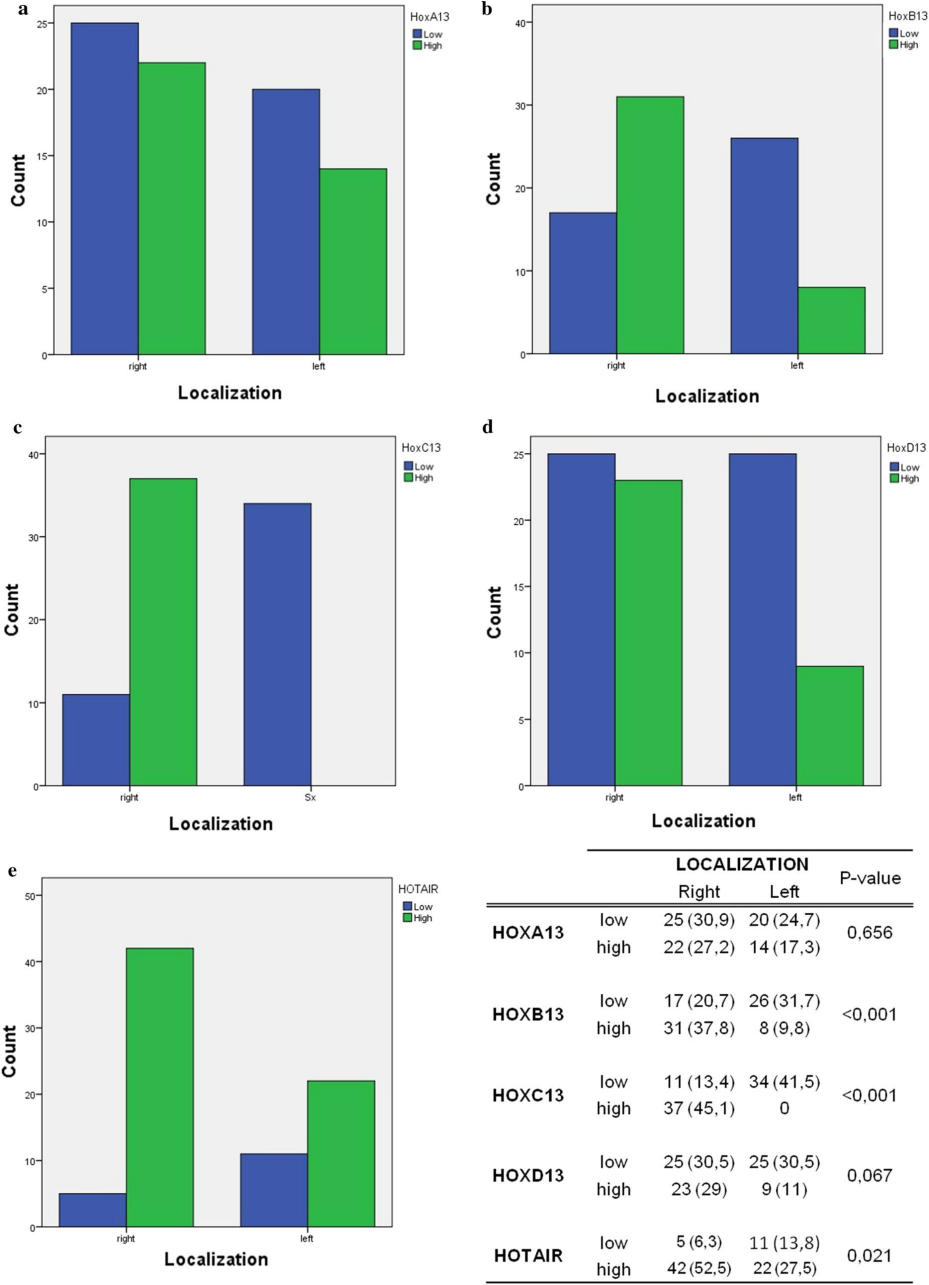
Graphical representation of paralogous 13 HOX genes and *HOTAIR* distribution in right/left location of CRC tumor samples: **a** histogram of *HOXA13* protein distribution; **b** histogram of *HOXB13* protein distribution; **c** histogram of *HOXC13* protein distribution; **d** histogram of *HOXD13* protein distribution; **e** histogram of *HOTAIR* mRNA distribution. Blue line identify samples with low expression and green line those with high expression

Regarding CRC localization, the distribution of HOX protein expression was very heterogeneous. Whereas *HOXA13* does not show statistically significant associations, both *HOXB13* and *HOXC13* showed a strong association with right location of CRC samples (p value ≤ 0.001) (Fig. 3). A strong statistical association between high *HOTAIR* expression and right location of CRC samples is also present (p value = 0.021) (Fig. 3).

### Relation between HOTAIR and paralogous 13 HOX genes

*HOXA13* appeared not correlated with *HOTAIR* expression, while a statistical association was detected between *HOXB13* (p value = 0.02) and *HOX C13* (p value = 0.042) expression and only a trend (p value = 0.063) with *HOXD13* expression (Table 2).

**Table 2:** Correlation between paralogous group 13 (*HOXA13, HOXB13, HOXC13, HOXD13*) and HOTAIR expression in CRC samples

	HOXA13		p-value	HOXB13		p-value	HOXC13		p-value	HOXD13		P-value
	Low	High		Low	High		Low	High		Low	High	
HOTAIR												
Low	23 (29,1)	22 (27,8)	0.37	29 (36,6)	16 (20)	0.024	29 (36,3)	16 (20)	0.042	32 (44,4)	13 (16,25)	0.063
High	21 (26,6)	13 (16,5)		13 (16,3)	22 (27,5)		14 (17,5)	21 (26,3)		17 (20,7)	18 (22,2)	

### CEA expression relation with lymph node metastasis status and paralogous 13 HOX genes and HOTAIR expression

We performed IHC evaluation of CEA on our CRC case series (Additional file 3: Figure S1). CEA showed a trend of statistical association with lymph nodes metastasis (0.086) (Additional file 4: Table S3), but none statistical association with HOTAIR and HOX proteins.

## Discussion

Colorectal carcinoma is the most common tumor of developed countries and is responsible for about 10% of deaths due to malignancy. Its incidence is increasing all over the world and in Europe each year 200,000 new cases are diagnosed. There is increasing evidence that the location of the primary tumor, distal and proximal position, has an important prognostic value in patients with colorectal cancer. A recent meta-analysis study that collected information on 143,746 patients clearly showed that patients with left-side tumors (descending colon, sigma, and rectum) survive longer than those with tumors originating on the proximal side (caecum and ascending colon) independent of stage, race, adjuvant chemotherapy [20]. It is already known that there are substantial biological and molecular differences between proximal and distal colorectal tumors, starting from the different embryonic origin, up to the different genetic instability and the frequency of specific mutations in the two different sites. Numerous studies have shown that proximal tumors are more often associated with microsatellite instability (MSI) and present further potentially deleterious mutations, including *KRAS*, *BRAF* and *PIK3CA* mutations [3, 4, 21]. The differences in the content of the lumen and of the bacterial flora between the left and right colon can also influence oncogenesis [22].

To deepen the understanding of the molecular mechanisms that determine the pathogenesis of the tumor on the left or right colon, in this study we decided to investigate the role of HOX genes belonging to the paralogous group 13 in a series of CRCs with different anatomical localization.

The HOX genes, in particular the posterior genes of the network, control the antero-posterior development of the intestine [16], and their aberrant expression has already been associated with colorectal carcinogenesis [17]. Our data showed the absence of expression of *HOXA13, HOXB13, HOXC13* and *HOXD13* in the normal mucosa, a slight increase in expression in the transitional mucosa, up to in many cases over-expressed in the tumor. In detail, *HOX A13* and *HOXD13* expression not show specific differences in the proximal/distal colon distribution and nor statistically significant associations with the clinical-pathological parameters of CRC patients. It is known that posterior HOX genes are expressed early during embryonic development of the intestine and represents during all embryogenic phases an important tissue-specific marker [16]. Although, numerous indications in the literature highlight their role in the pathogenesis and progression of various tumors associated with the digestive system, in particular in hepatocellular carcinoma [8], and gastric tumors [23] our data do not suggest a fundamental role of *HOX A13* and *HOXD13* genes in colon cancer evolution.

Regarding *HOX B13* expression, it strongly correlates with lymph nodes metastasis and showed a prevalent expression in CRC samples located in the right side. In the literature, while the role of *HOX B13* is very well detailed in the pathogenesis in particular of prostate tumors [24] and of the breast and ovarian cancers [25, 26], little information is present regarding its role in the neoplastic evolution of the colon. In only a single study on CRC cell models downregulation of *HOX B13* has been described [27]. More recently, in line with our data, a gene array study showed a prevalence of its expression in the proximal localization of CRCs [28].

The same trend has been highlighted for *HOXC13,* correlated with lymph nodes metastasis and with a prevalent expression in the right CRCs samples. *HOX C13* plays a fundamental role in the control of cell proliferation and tumorigenesis in cellular models of colorectal adenocarcinoma (SW480), breast carcinoma (MCF7), prostate cancer (PC3ML), cervical (HeLa), and renal (HEK293) [29].

As the expression of the HOX genes is under the control of a series of non-coding RNA that co-localize within the HOX loci [14], we have focused our attention in particular on the long non-coding RNA *HOTAIR* which controls in trans the expression of the locus D genes and genes associated with metastatic progression [15].

Our data have shown the expression in situ of the *HOTAIR* on tissue samples, that appears silent in healthy tissue, and becomes overexpressed in the neoplastic tissue, validating the previous studies carried out with molecular methods [30].

*HOTAIR* expression appears strongly statistically associated with T stage and lymph nodes metastasis and shows an higher expression in the tumors located in the right/proximal colon. Moreover, a direct correlation between *HOTAIR* and *HOX B13* and *HOX C13* has been highlighted in our case series.

Our data are in line with the previous studies carried out on different solid tumors, in which *HOTAIR* primarily appeared as marker of lymph node metastasis [18, 31, 32]. In support of this hypothesis, 2 meta-analysis studies have analyzed the large amount of data produced in recent years, showing that high *HOTAIR* expression in tumor tissue is strongly correlated with lymph node metastasis [33, 34]. In breast cancer patients high circulating *HOTAIR* level is also associated with lymph node metastasis [35].

On our CRC case series we have also performed an immunohistochemical analysis of CEA to establish its potential relation with lymph nodes metastasis status, as previously reported [36], and the expression of HOTAIR and HOX proteins. However, we do not show any strong statistical association nor with metastatic disease nor with the expression of selected markers.

## Conclusions

In conclusion, our data highlighted an involvement of *HOXB13, HOXC13* and *HOTAIR* in proximal colon cancers pathogenesis and in lymph nodes metastasis progression, highlighting the main role of these markers in prognosis of colon cancer patients. Beyond additional elements for the understanding of molecular mechanisms underlying the colon tumorigenesis we can speculate that our data may represent an important element for the development of potential therapies targeted against the activities of HOX genes. In fact, several studies showed the possibility of targeting the post-translational interaction between HOX proteins and their PBX cofactors, through the short peptide HXR9 that is able to bind a conserved six-amino acid sequence in the HOX proteins [37]. These in vitro and in vivo data supported more and more the therapeutic potential of inhibiting HOX/PBX dimer formation in cancer [38].

Additionally, the role of *HOTAIR* in the modulation of drug resistance mechanisms has been extensively described for several solid tumors [39–41], and since lncRNAs are molecules with high stability in biological fluids the detection of *HOTAIR* in the blood of CRC patients could represent an useful prognostic tool also for the prediction and monitoring of therapeutic response.

## Additional files

**Additional file 1: Table S1.** Cut-off expression of HOXA13, HOXB13, HOXC13 and HOTAIR.

**Additional file 2: Table S2.** Main clinic-pathological features of CRC patients.

**Additional file 3: Figure S1.** CEA expression in colon tissue samples: a) low; b) medium and c) high IHC staining (×40).

**Additional file 4: Table S3.** Correlation between CEA expression and lymph nodes metastasis status.

## Abbreviations

LncRNA: long non coding RNA
HOTAIR: hox transcript antisense intergenic RNA
CRC: colorectal cancer
MSI: microsatellite instability
KRAS: kirsten rat sarcoma viral oncogene homolog
BRAF: V-raf murine sarcoma viral oncogene homolog b1
PIK3CA: phosphatidylinositol-4,5-bisphosphate 3-kinase catalytic subunit alpha
WHO: World Health Organization Classification of Tumours
H&E: Hematoxylin & Eosin
