# Supplementary material for: Posterior HOX genes and HOTAIR expression in the proximal and distal colon cancer pathogenesis
Source: J Transl Med. 2018 Dec 12;16:350. doi: 10.1186/s12967-018-1725-y (PMC6292169; doi:10.1186/s12967-018-1725-y)
Supplement: Supplementary file 1 — Additional file 1: Table S1. Cut-off expression of HOXA13, HOXB13, HOXC13 and HOTAIR. [file 12967_2018_1725_MOESM1_ESM.docx]

|  | Expression | |
| --- | --- | --- |
|  | low | high |
| HOXA13 | ≤ 50 | 50+ |
| HOXB13 | ≤ 50 | 50+ |
| HOXC13 | ≤ 2 | 2+ |
| HOXD13 | ≤ 50 | 50+ |
| HOTAIR | ≤ 30 | 30+ |
|  |  |  |

**Additional file 1: Table S1**
