# Supplementary material for: Posterior HOX genes and HOTAIR expression in the proximal and distal colon cancer pathogenesis
Source: J Transl Med. 2018 Dec 12;16:350. doi: 10.1186/s12967-018-1725-y (PMC6292169; doi:10.1186/s12967-018-1725-y)
Supplement: Supplementary file 2 — Additional file 2: Table S2. Main clinic-pathological features of CRC patients. [file 12967_2018_1725_MOESM2_ESM.docx]

|  |  | **N°** |
| --- | --- | --- |
| **Age** | <65 | 36 / 82 |
|  | >65 | 46 / 82 |
| **Gender** | M | 44 / 82 |
|  | F | 38 / 82 |
| **Localization** | Dx | 48 / 82 |
|  | Sx | 34 / 82 |
| **T** | 0 | 3 / 82 |
|  | 1 | 6 / 82 |
|  | 2 | 25 / 82 |
|  | 3 | 42 / 82 |
|  | 4 | 6 / 82 |
| **N** | 0 | 48 / 74 |
|  | 1 | 16 / 74 |
|  | 2 | 9 / 74 |
|  | 3 | 1 / 74 |
| **Grade** | G1 | 3 / 82 |
|  | G2 | 74 / 82 |
|  | G3 | 5 / 82 |

Additional file 2: Table S2
