# Supplementary figures and images for: Posterior HOX genes and HOTAIR expression in the proximal and distal colon cancer pathogenesis
Source: J Transl Med. 2018 Dec 12;16:350. doi: 10.1186/s12967-018-1725-y (PMC6292169; doi:10.1186/s12967-018-1725-y)

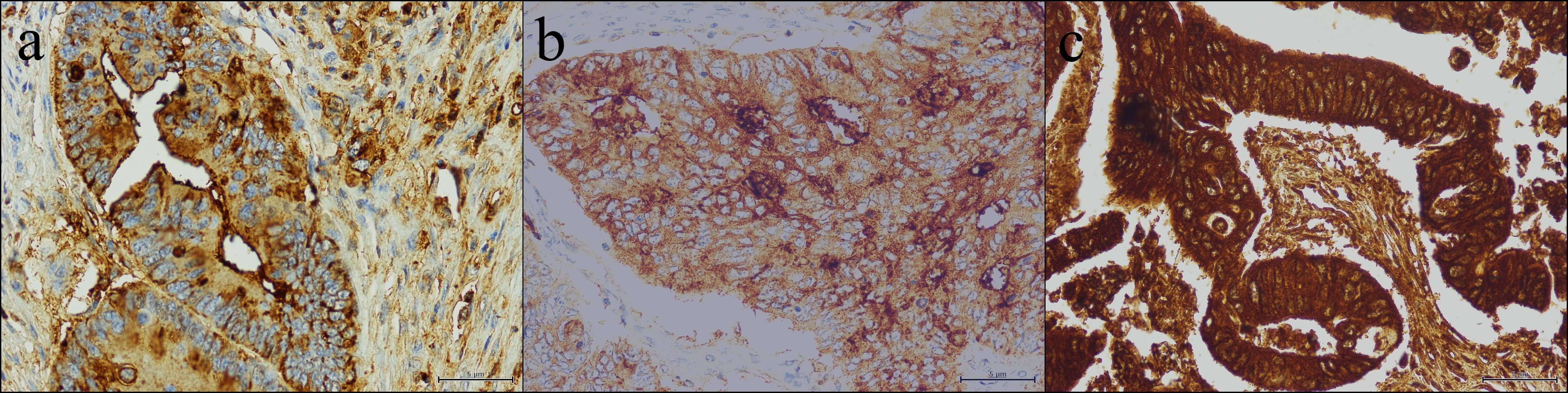

Supplement: Supplementary file 3 — Additional file 3: Figure S1. CEA expression in colon tissue samples: a) low; b) medium and c) high IHC staining (×40). [file 12967_2018_1725_MOESM3_ESM.jpg]
