# Supplementary material for: Posterior HOX genes and HOTAIR expression in the proximal and distal colon cancer pathogenesis
Source: J Transl Med. 2018 Dec 12;16:350. doi: 10.1186/s12967-018-1725-y (PMC6292169; doi:10.1186/s12967-018-1725-y)
Supplement: Supplementary file 4 — Additional file 4: Table S3. Correlation between CEA expression and lymph nodes metastasis status. [file 12967_2018_1725_MOESM4_ESM.docx]

|  | Intensity score | N0 | N+ | P-value |
| --- | --- | --- | --- | --- |
| CEA | 1+ | 9 | 0 | 0,086 |
|  | 2+ | 25 | 11 |  |
|  | 3+ | 14 | 14 |  |
|  |  |  |  |  |

**Additional file 3: Table S3**
